# Myogenic Progenitor Cell Lineage Specification by CRISPR/Cas9-Based Transcriptional Activators

**DOI:** 10.1016/j.stemcr.2020.03.026

**Published:** 2020-04-23

**Authors:** Jennifer B. Kwon, Ashish Vankara, Adarsh R. Ettyreddy, Joel D. Bohning, Charles A. Gersbach

**Affiliations:** 1 University Program in Genetics and Genomics, Duke University Medical Center, Durham, NC 27710, USA; 2 Center for Advanced Genomic Technologies, Duke University, Durham, NC 27708, USA; 3 Department of Biomedical Engineering, Duke University, Durham, NC 27708, USA; 4 Department of Surgery, Duke University Medical Center, Durham, NC 27710, USA

**Keywords:** pluripotent, differentiation, CRISPR, epigenome editing, myoblasts, satellite cells, myogenesis, iPSCs

## Abstract

Engineered CRISPR/Cas9-based transcriptional activators can potently and specifically activate endogenous fate-determining genes to direct differentiation of pluripotent stem cells. Here, we demonstrate that endogenous activation of the *PAX7* transcription factor results in stable epigenetic remodeling and differentiates human pluripotent stem cells into skeletal myogenic progenitor cells. Compared with exogenous overexpression of *PAX7* cDNA, we find that endogenous activation results in the generation of more proliferative myogenic progenitors that can maintain PAX7 expression over multiple passages in serum-free conditions while preserving the capacity for terminal myogenic differentiation. Transplantation of human myogenic progenitors derived from endogenous activation of *PAX7* into immunodeficient mice resulted in a greater number of human dystrophin^+^ myofibers compared with exogenous *PAX7* overexpression. RNA-sequencing analysis also revealed transcriptome-wide differences between myogenic progenitors generated via CRISPR-based endogenous activation of *PAX7* and exogenous *PAX7* cDNA overexpression. These studies demonstrate the utility of CRISPR/Cas9-based transcriptional activators for controlling cell-fate decisions.

## Highlights

• CRISPR activation generates myogenic progenitors from human iPSCs/ESCs
• Activation of endogenous *PAX7* leads to autonomously maintained gene expression
• Endogenous activation outperforms cDNA expression in generating a stable phenotype
• CRISPRa myogenic progenitors engraft and regenerate muscle fibers *in vivo* in mice

## Graphical Abstract

**Figure:**
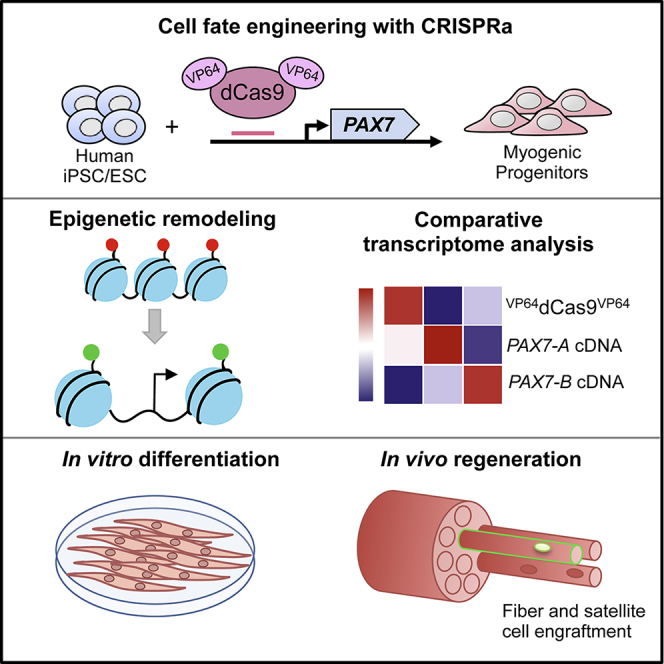

## Teaser

In this article, Gersbach and colleagues use a CRISPR/Cas9-based transcriptional activator to differentiate hPSCs into myogenic progenitor cells that can differentiate *in vitro* and participate in regeneration *in vivo*. They demonstrate stable epigenetic remodeling of the *PAX7* locus and uncover transcriptome-wide differences that result from endogenous versus exogenous *PAX7* expression.

## Introduction

Human pluripotent stem cells (hPSCs) are a promising cell source for regenerative medicine, disease modeling, and drug discovery in pathologies of muscle disease. Directed differentiation of hPSCs into skeletal muscle cells can be achieved via stepwise small-molecule-based protocols (Barberi et al., 2007, Chal et al., 2015, Shelton et al., 2014, Wu et al., 2018) or ectopic expression of transgenes (Darabi et al., 2012, Darabi et al., 2008, Tedesco et al., 2012). While having the benefit of being transgene free, small-molecule-based protocols tend to be relatively lengthy and inefficient, and lack the scalability required for cell therapy or drug-screening applications (Jiwlawat et al., 2018). Transgene-based approaches rely on overexpression of key transcription factors, including PAX3, PAX7, and MYOD. These protocols are relatively efficient in yielding populations of myogenic cells, and they do so more rapidly than transgene-free methods (Kodaka et al., 2017). Generation of satellite cells—the skeletal muscle stem cell population—is particularly appealing for myogenic cell therapies. Although satellite cells can robustly regenerate damaged muscles *in vivo*, they cannot be isolated and expanded *ex vivo* without relinquishing their stemness, resulting in loss of engraftment capabilities (Montarras, 2005). Consequently, the generation of functional PAX7^+^ satellite cells from hPSCs has been attempted by pairing various differentiation protocols with exogenous PAX7 cDNA overexpression (Darabi et al., 2012, Kim et al., 2017, Rao et al., 2018). Here, we explore an alternative strategy of generating myogenic progenitor cells via activation of the endogenous *PAX7* gene.

Advances in genome-engineering technologies have established the type II clustered regularly interspaced short palindromic repeats (CRISPR)/Cas9 system as a programmable transcriptional regulator capable of targeted activation or repression of endogenous genes. Mutations to the catalytic residues of the Cas9 protein results in a nuclease-deactivated Cas9 (dCas9) that can be fused to various effector domains to exert their function on precise genomic loci defined by the guide RNA (gRNA) (Gilbert et al., 2013, Jinek et al., 2012). For example, fusion of dCas9 to the transactivation domain VP64 can potently activate genes in their native chromosomal context when gRNAs are designed at target gene promoters (Maeder et al., 2013, Perez-Pinera et al., 2013). In contrast to ectopic expression of transgenes, activation of endogenous genes facilitates chromatin remodeling and induction of autonomously maintained gene networks (Black et al., 2016, Chakraborty et al., 2014, Liu et al., 2018a). Targeting endogenous genes can also capture the full complexity of transcript isoforms, mRNA localization, and other effects of non-coding regulatory elements, which may be critical for proper cellular reprogramming. We and several other groups have demonstrated cellular reprogramming with CRISPR/Cas9-based transcriptional regulators in the context of somatic cell reprogramming (Balboa et al., 2015, Black et al., 2016, Chakraborty et al., 2014, Liu et al., 2018a, Weltner et al., 2018) as well as directed differentiation of pluripotent stem cells (PSCs) into various cell types (Balboa et al., 2015, Chavez et al., 2015, Kearns et al., 2014, Liu et al., 2018b). However, there has not yet been a demonstration of differentiation of hPSCs with CRISPR/Cas9-based transcriptional activators to generate cells capable of *in vivo* transplantation, engraftment, and tissue regeneration.

In this study, we use ^VP64^dCas9^VP64^ to activate the endogenous gene encoding the transcription factor PAX7 in both human embryonic stem cells (ESCs) and induced PSCs (iPSCs) to direct differentiation into skeletal muscle progenitors. We demonstrate the derivation of functional skeletal muscle progenitor cells that can be induced to differentiate *in vitro* and can also participate in regeneration of damaged muscles *in vivo* when transplanted into mice. We also demonstrate stable epigenetic remodeling of the *PAX7* locus and uncover transcriptome-wide differences that result from endogenous versus exogenous *PAX7* expression. These results establish CRISPR-based activation of gene networks governing progenitor cell specification as a potential strategy for cell therapy and regenerative medicine.

## Results

### Developing Conditions for ^VP64^dCas9^VP64^-Mediated Endogenous *PAX7* Activation in hPSCs

During embryonic differentiation, PAX7 and its paralog PAX3 specify myogenic cells within the paraxial mesoderm. Differentiation of hPSCs into paraxial mesoderm cells can be initiated by CHIR99021, a GSK3 inhibitor (Tan et al., 2013). Two human pluripotent stem cell lines, H9 ESCs and DU11 iPSCs, were used for differentiation studies. For targeted gene activation, we used the dCas9 with the VP64 domain fused to both the N and C termini (^VP64^dCas9^VP64^), which we previously showed to be ∼10-fold more potent than a single VP64 fusion (Black et al., 2016, Chakraborty et al., 2014). To test the efficacy of ^VP64^dCas9^VP64^-mediated activation of *PAX7*, we designed eight gRNAs spanning −490 to +158 bp relative to the transcription start site (TSS) of the human *PAX7* gene (Figure S1A). H9 ESCs stably expressing ^VP64^dCas9^VP64^ were differentiated into paraxial mesoderm cells with addition of CHIR99021 in E6 medium for 2 days, as previously described (Shelton et al., 2014). Cells were transfected with the individual gRNAs and samples were harvested 6 days later for gene-expression analysis using qRT-PCR. Four out of the eight gRNAs significantly upregulated *PAX7* compared with mock transfected cells (Figure S1B). In a second screen, we packaged the four individual gRNAs that performed best in the transfection experiment into lentiviruses to achieve more stable and robust expression. Cells were harvested at 8 days post transduction. gRNA #4 was identified as the most potent gRNA and was used for subsequent studies (Figure S1C).

### ^VP64^dCas9^VP64^-Mediated Differentiation of hPSCs into Myogenic Progenitor Cells

**Figure 1:**
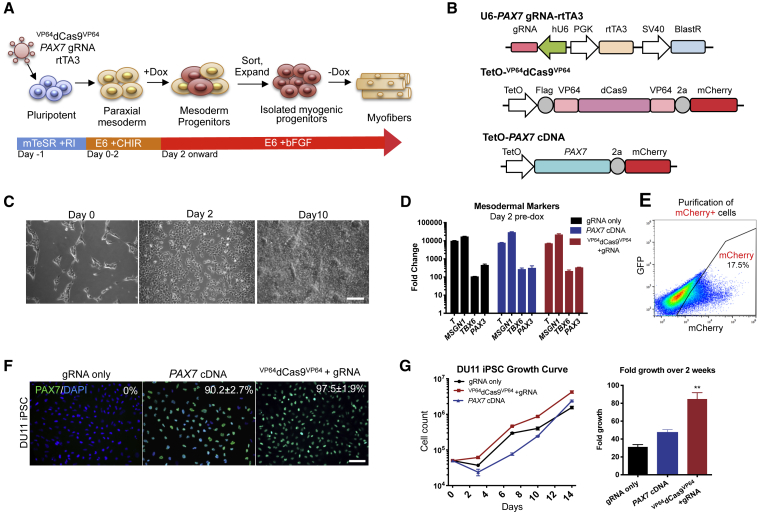
Generation of Myogenic Progenitors from hPSCs via ^VP64^dCas9^VP64^-Mediated Activation of Endogenous *PAX7* (A) Schematic of hPSC myogenic differentiation with small molecules and lentiviral activation of *PAX7*. (B) The lentiviral constructs used for the gRNA and inducible ^VP64^dCas9^VP64^ and *PAX7* cDNA expression. (C) Representative phase-contrast images showing morphological changes during the first 10 days of differentiation. Scale bar, 200 μm. (D) RNA was harvested at day 0 and day 2 for qRT-PCR analysis of mesodermal markers. Results are expressed as fold change over day 0 (mean ± SEM, n = 3 independent replicates). (E) Representative fluorescence-activated cell sorting (FACS) plot at day 14 when ^VP64^dCas9^VP64^-2a-mCherry^+^ cells were sorted for expansion. (F) Representative immunostaining of PAX7 at 5 days post sort. Scale bar, 100 μm. (G) Growth of purified myogenic progenitors derived from iPSC differentiation during post-sort expansion phase was monitored over 2 weeks. Fold-growth over 2 weeks was significantly greater in ^VP64^dCas9^VP64^-treated cells compared with *PAX7* cDNA-treated cells. p values were determined by one-way ANOVA followed by Tukey's post hoc test (mean ± SEM, n = 3 independent replicates).

Next, we tested the hypothesis that endogenous *PAX7* activation in paraxial mesoderm cells would be sufficient for generating myogenic progenitor cells (MPCs) with the potential to differentiate into myotubes *in vitro* (Figure 1A). Prior to differentiation, hPSCs were transduced with a lentivirus expressing the *PAX7* promoter-targeting gRNA, a reverse tetracycline transactivator (rtTA), and a blasticidin resistance gene. Cells were selected with blasticidin for stable expression of the vector and then transduced with an additional lentivirus encoding either doxycycline (dox)-inducible ^VP64^dCas9^VP64^ or the *PAX7* cDNA, which also included a co-transcribed mCherry reporter gene (Figure 1B). hPSCs were differentiated with CHIR99021 for 2 days and then maintained in E6 medium with dox and fibroblast growth factor 2 (FGF2) to support MPC proliferation (Figure 1C) (Pawlikowski et al., 2017). Addition of CHIR99021 induced paraxial mesodermal differentiation, as indicated by high levels of pan-mesoderm marker Brachyury (*T*), paraxial mesoderm markers *MSGN1* and *TBX6*, and pre-myogenic mesoderm marker *PAX3* at the mRNA level (Figure 1D). Transduced cells were sorted based on mCherry expression after 2 weeks of growth (Figure 1E). mCherry^+^ cells accounted for ∼20% of cells transduced with ^VP64^dCas9^VP64^ compared with ∼50% with *PAX7* cDNA transduced cells. This is likely due to the larger size of ^VP64^dCas9^VP64^ vector compared with the *PAX7* cDNA vector (7.9 kb between long terminal repeats versus 4.9 kb) resulting in reduced lentiviral titers. These purified MPCs were maintained in serum-free E6 medium supplemented with dox and FGF2 and passaged when cells reached ∼80% confluency. Sorted cells demonstrated high purity of PAX7^+^ cells in both the endogenous-activated cells and exogenous cDNA-expressing cells when protein expression was assessed by immunofluorescence staining 5 days after sorting (Figures 1F and S2A). ^VP64^dCas9^VP64^-treated iPSCs and ESCs both demonstrated notable expansion potential, averaging 85-fold and 95-fold increase in cell number, respectively, over the 2 weeks after purification. Furthermore, the growth potential of these cells outperformed the *PAX7* cDNA-overexpressing cells (Figures 1G and S2B).

### Characterization of MPCs Derived from Endogenous or Exogenous *PAX7* Expression

**Figure 2:**
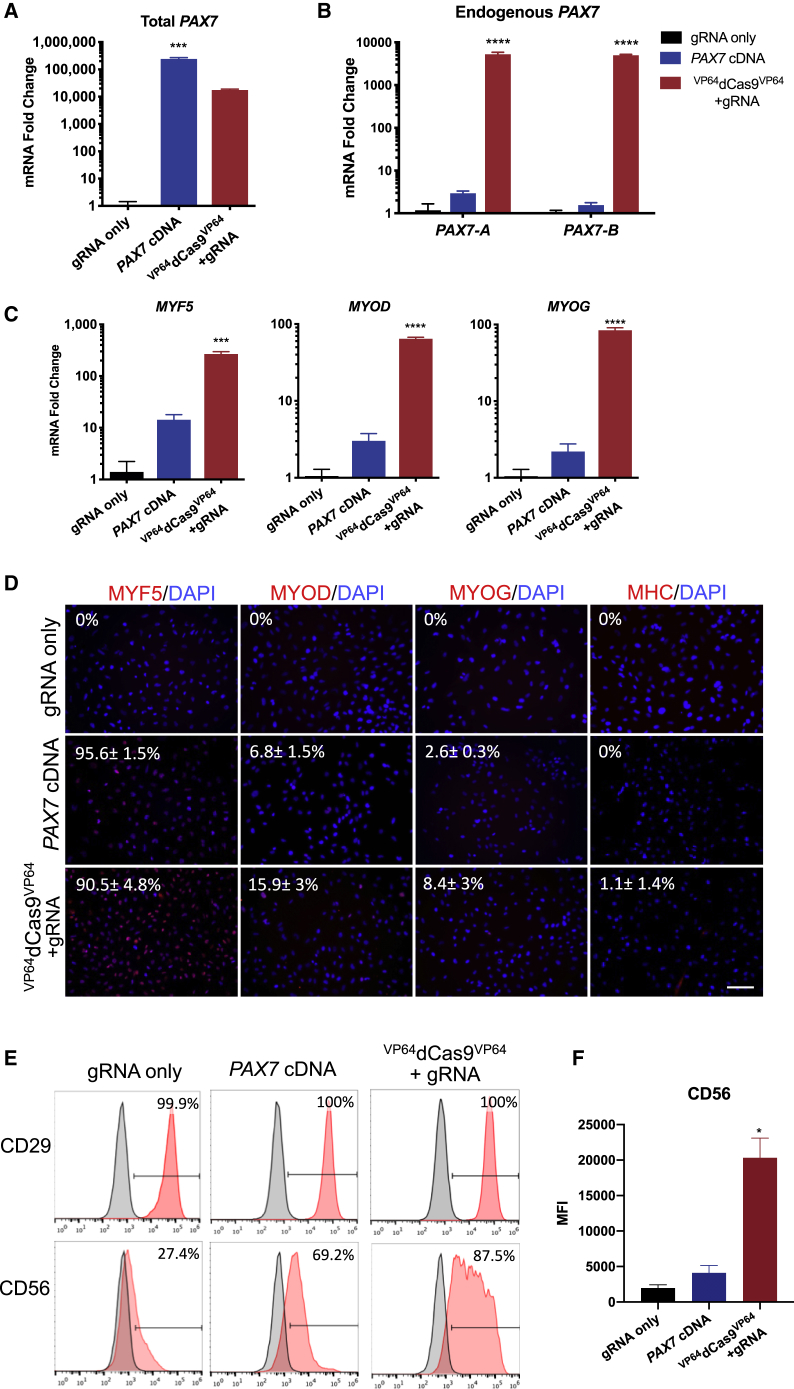
Characterization of Myogenic Progenitors Derived from iPSCs via ^VP64^dCas9^VP64^-Mediated Activation of Endogenous *PAX7* or Exogenous *PAX7* cDNA Expression (A) Relative amounts of total *PAX7* mRNA was determined by qRT-PCR using primers complementary to sequences present in the gene body. (B) Endogenous *PAX7* mRNA was detected using primers complementary to sequences in the 3′ UTR of either isoform *PAX7-A* or *PAX7-B*. (C) The mRNA expression levels of myogenic markers *MYF5*, *MYOD*, and *MYOG* during the expansion phase. (D) Immunofluorescence staining of early and mature myogenic markers MYF5, MYOD, and MYOG, and myosin heavy chain (MHC). (E) Representative FACS analysis of CD29 and CD56 surface marker expression during the expansion phase. (F) Mean fluorescence intensity (MFI) of CD56 staining intensity across treatments. All p values were determined by one-way ANOVA followed by Tukey's post hoc test (mean ± SEM, n = 3 independent replicates).

*PAX7* mRNA levels were assessed by qRT-PCR during the proliferation phase 5 days after sorting. *PAX7* mRNA from the endogenous chromosomal locus could be discriminated from total *PAX7* mRNA, made from either the lentivirus or endogenous chromosomal locus, using distinct primer pairs. While overexpression of *PAX7* cDNA resulted in more total *PAX7* mRNA (Figures 2A and S2C), robust detection of any endogenous *PAX7* isoform was only observed in ^VP64^dCas9^VP64^-treated cells (Figures 2B and S2D). The human *PAX7* gene encodes multiple isoforms of which differential sequences have been identified, but unique biological functions remain unclear (Barr et al., 1999, Lamey et al., 2004, Vorobyov and Horst, 2004). Differential transcriptional termination in either exon 8 or exon 9 yields *PAX7-A* and *PAX7-B* isoforms, respectively. The differences in the 3′ ends of these transcripts allow for differential detection with unique qRT-PCR primers.

Downstream myogenic regulatory factors *MYF5*, *MYOD*, and *MYOG* were also detected at the mRNA level by qRT-PCR (Figures 2C and S2E). At the protein level, the majority of cells in both endogenous and exogenous PAX7-expressing cells co-expressed the activated satellite cell marker, MYF5 (>90%). The myoblast marker, MYOD, was expressed higher in cells expressing endogenous *PAX7* compared with exogenous *PAX7* cDNA, at 15.9% and 6.8%, respectively. Mature myogenic markers MYOG and myosin heavy chain (MHC) were detectable at a low level in some of the cells (Figure 2D).

Human satellite cells co-express PAX7 with CD29 and CD56 surface markers (Xu et al., 2015). At approximately 10 days after sorting, we assessed our MPCs for CD29 and CD56 expression and found that 100% of cells in all groups expressed CD29, independent of PAX7 expression. We found that CD56 expression was more contingent on PAX7 expression, with only 27.4% of cells expressing CD56 in the gRNA-only group, compared with 69.2% and 87.5% of cells in the *PAX7* cDNA-treated and ^VP64^dCas9^VP64^-treated groups, respectively (Figures 2E and S2F). Assessment of mean fluorescence intensity of CD56 staining also revealed that the average CD56 expression level per cell was significantly higher in the ^VP64^dCas9^VP64^-treated group (Figures 2F and S2G).

### Transplantation of ^VP64^dCas9^VP64^-Generated Myogenic Progenitors into Immunodeficient Mice Demonstrates *In Vivo* Regenerative Potential

**Figure 3:**
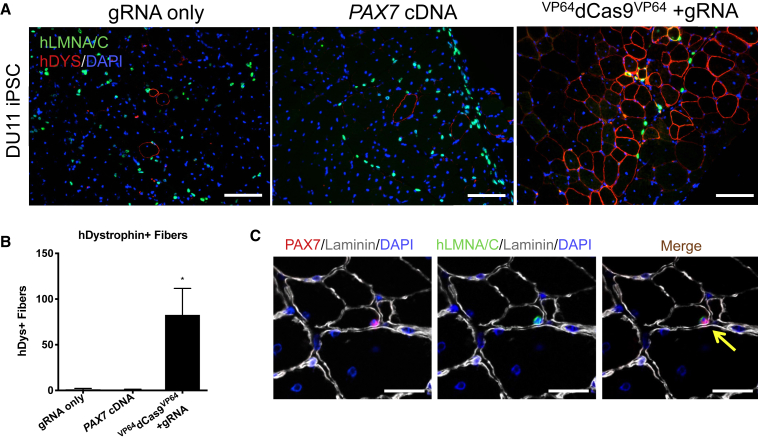
Transplantation of ^VP64^dCas9^VP64^-Generated Myogenic Progenitors into Immunodeficient Mice Demonstrates *In Vivo* Regenerative Potential (A) Detection of human-derived fibers in ^VP64^dCas9^VP64^-treated cells 1 month after intramuscular injection of 5 × 10^5^ differentiated iPSCs into NSG mice pre-injured with BaCl_2_. Sections are stained with human-specific dystrophin and lamin A/C antibodies to mark donor-derived fibers and nuclei. Scale bars, 100 μm. (B) Quantification of human dystrophin^+^ fibers in the section with highest number of dystrophin^+^ fibers in each muscle. ^∗^p < 0.05 determined by Student's t test compared with control (mean ± SEM, n = 3 mice). (C) Identification of donor-derived satellite cells expressing PAX7 and human-specific lamin A/C, and residing adjacent to the basal lamina as indicated by laminin staining. Scale bars, 25 μm.

We next determined whether MPCs derived from ^VP64^dCas9^VP64^-mediated *PAX7* activation possess *in vivo* regenerative potential. Cells that had been expanded and passaged three times post sort were transplanted into the tibialis anterior (TA) of immunodeficient NOD.SCID.gamma (NSG) mice that were pre-injured with barium chloride (BaCl_2_) to create a regenerative microenvironment (Hall et al., 2010). Twenty-four hours after injury, mice were injected with 500,000 cells treated with gRNA only, *PAX7* cDNA overexpression, or ^VP64^dCas9^VP64^-mediated endogenous *PAX7* activation. One month after transplantation, muscles were harvested and evaluated for engraftment by immunostaining with human-specific dystrophin and lamin A/C antibodies. Human nuclei were detected by lamin A/C staining in all three conditions; however, only the endogenous *PAX7*-activated group demonstrated consistent presence of human dystrophin-expressing myofibers (Figures 3A and S2I). The number of human dystrophin^+^ fibers was quantified across three mice per condition by counting sections with most abundant human dystrophin^+^ fibers within each sample (Figure 3B). We also investigated whether transplanted cells could seed the satellite cell niche. Immunostaining for PAX7, human lamin A/C, and laminin was performed to demarcate satellite cells of human origin. PAX7 and human lamin A/C double-positive cells residing under the basal lamina were identified only in muscle transplanted with ^VP64^dCas9^VP64^-activated MPCs (Figures 3C and S2J).

### Induction of Endogenous *PAX7* Expression Is Sustained after Multiple Passages and Dox Withdrawal

**Figure 4:**
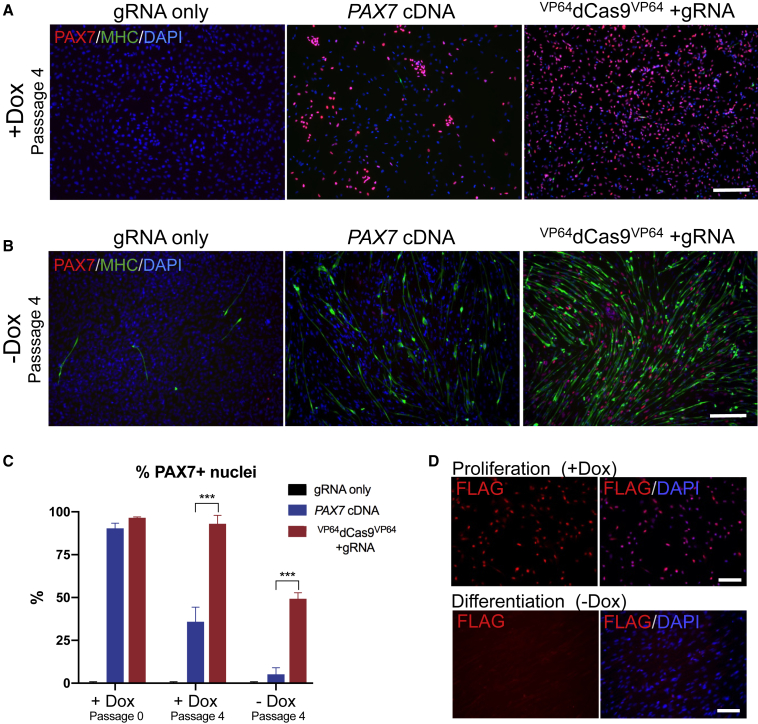
Induction of Endogenous PAX7 Expression Is Sustained after Multiple Passages and Dox Withdrawal (A) Representative immunostaining of PAX7 and MHC in differentiated iPSCs after four passages in the presence of dox. Scale bar, 200 μm. (B) Representative immunostaining of PAX7 and myosin heavy chain (MHC) after inducing differentiation by dox withdrawal for 7 days. Scale bar, 200 μm. (C) Quantification of PAX7^+^ nuclei after no passages and after an average of four additional passages with dox or after dox withdrawal (mean ± SEM, n = 3 independent experiments). (D) Representative immunostaining of the FLAG epitope for ^VP64^dCas9^VP64^ after dox withdrawal for 7 days. Scale bars, 100 μm.

During expansion of sorted cells, we noticed a significant decrease in PAX7^+^ cells in the cDNA overexpression group after an average of four passages spanning an average of 32 days in three independent experiments. Although the initial number of cells expressing PAX7 protein was >90% at 5 days post sort, quantification of PAX7^+^ nuclei following approximately four passages after initial flow sorting revealed that only a minority of cells (35.8%) expressed PAX7 protein despite maintenance in dox during the expansion period. Conversely, a large majority (93%) of endogenously activated *PAX7* cells retained PAX7 protein expression without precocious differentiation across multiple passages (Figures 4A and 4C). As indicated by lack of MHC^+^ cells, depletion of PAX7^+^ cells in the cDNA overexpression group did not correspond to the adoption of a myogenic fate (Figure 4A). We postulated that this may be due to high levels of PAX7 protein hindering cell proliferation, allowing for cells that have silenced the promoter or contaminating cells from the sort to overtake the cell population. Consistent with this possibility, *Pax7* cDNA overexpression has been previously implicated in inducing cell-cycle exit without commitment to myogenic differentiation (Olguin and Olwin, 2004). Interestingly, a previously published study also observed this phenomenon of PAX7 loss over multiple passages when using a tet-inducible *PAX7* cDNA overexpression system. That study required amendment of the serum-free differentiation protocol to media conditions containing highly mitogenic 20% fetal calf serum to improve retention of PAX7 protein expression in cDNA-overexpressing cells (Rao et al., 2018).

Differentiation of pre-myogenic cells was induced by withdrawing dox when cells reached 100% confluency. Abundant MHC^+^ myofibers were observed in ^VP64^dCas9^VP64^-treated cells (Figures 4B and S2H). Interestingly, 50% of cells remained PAX7^+^ in the ^VP64^dCas9^VP64^ treated group despite 1 week without dox. In contrast, only 5.2% of cells in the *PAX7* cDNA-treated group demonstrated PAX7 protein expression after 1 week without dox (Figure 4C). Staining for the FLAG epitope confirmed the absence of ^VP64^dCas9^VP64^ in differentiated cells at this time point (Figure 4D).

### ^VP64^dCas9^VP64^ Leads to Sustained PAX7 Expression and Stable Chromatin Remodeling at Target Locus

**Figure 5:**
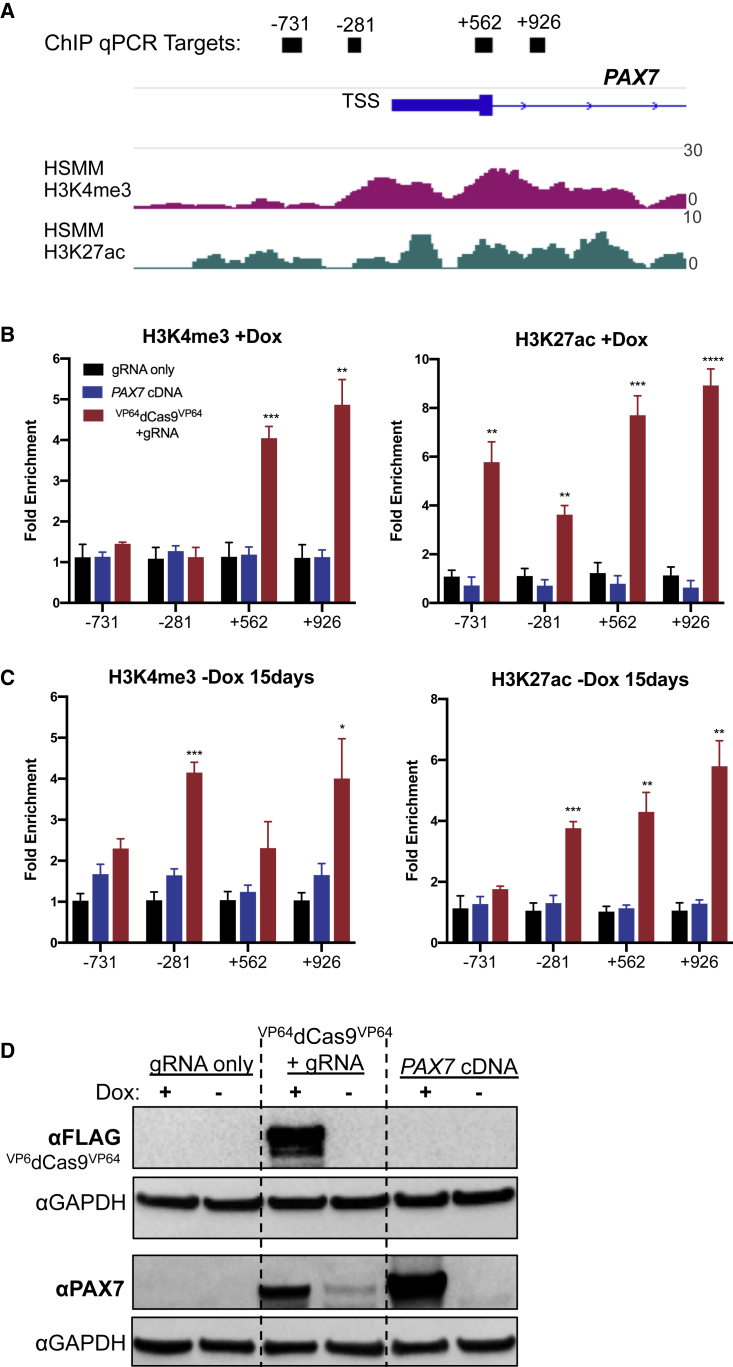
^VP64^dCas9^VP64^ Leads to Sustained PAX7 Expression and Stable Chromatin Remodeling at Target Locus (A) Human genomic track spanning the PAX7 TSS region depicting H3K4me3 and H3K27ac enrichment in human skeletal muscle myoblast (HSMM). Data from ENCODE (GEO: GSM733637; GSM733755). Black bars indicate ChIP-qPCR target regions. (B) Targeted activation of endogenous *PAX7* induced significant enrichment of H3K4me3 and H3K27ac around the TSS in the presence of dox in proliferation conditions. (C) Enrichment of histone marks is sustained after 15 days in the absence of dox in proliferation conditions (mean ± SEM, n = 3 independent replicates). (D) An N-terminal FLAG epitope tag was used to verify depletion of ^VP64^dCas9^VP64^ after 15 days without dox, which was concomitant with sustained PAX7 protein expression.

We hypothesized that epigenetic remodeling of the endogenous *PAX7* promoter was allowing cells to autonomously upregulate *PAX7* without the continued presence of ^VP64^dCas9^VP64^. To investigate this, we performed chromatin immunoprecipitation (ChIP)-qPCR on cells during dox administration and at 15 days after dox withdrawal. Cells were analyzed at day 30 of differentiation for the +dox condition and then expanded and passaged three more times over 15 days in the absence of dox. We used ChIP-seq data generated as part of the Encyclopedia of DNA Elements (ENCODE) Project to identify histone modifications enriched at the transcriptionally active *PAX7* in human skeletal muscle myoblasts, including H3K4me3 and H3K27ac (Figure 5A). Four qPCR primers were designed to tile regions −731 to +926 bp relative to the *PAX7* TSS. ChIP-qPCR of +dox conditions demonstrated significant enrichment of H3K4me3 and H3K27ac at the endogenous *PAX7* locus only in response to ^VP64^dCas9^VP64^ treatment (Figure 5B). Furthermore, these histone modifications were maintained for 15 days after dox withdrawal (Figure 5C). To ensure that there was no leaky expression of ^VP64^dCas9^VP64^ after dox removal, we performed a western blot for the FLAG epitope tag and were unable to detect ^VP64^dCas9^VP64^ 15 days after dox removal (Figure 5D). Conversely, PAX7 was still detectable by western blot in the absence of ^VP64^dCas9^VP64^, corresponding to the ChIP-qPCR enrichment of active histone marks.

### Identification of Endogenous versus Exogenous *PAX7*-Induced Global Transcriptional Changes

**Figure 6:**
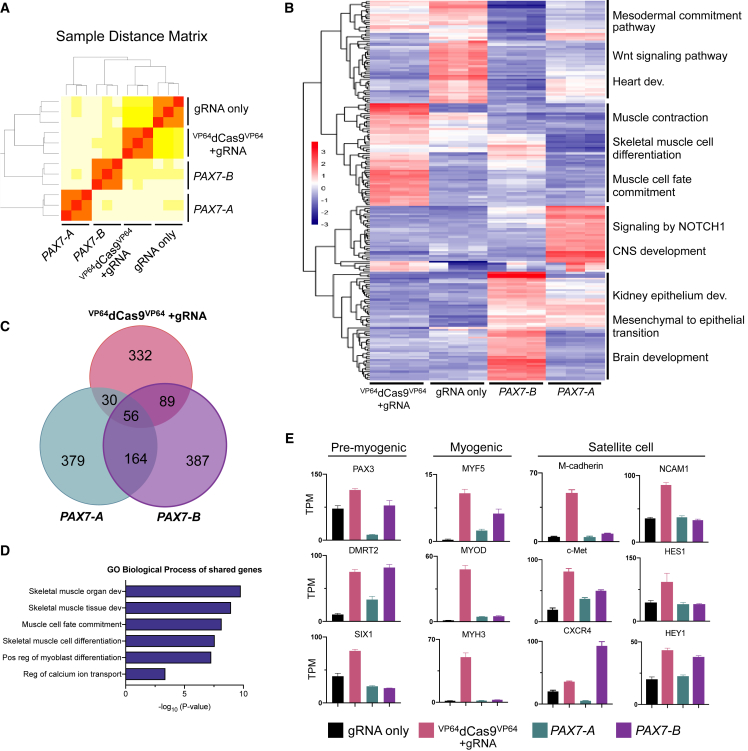
Identification of Endogenous versus Exogenous *PAX7*-Induced Global Transcriptional Changes (A) Expression heatmap of sample-to-sample distances in the matrix using the whole gene-expression profiles among the four groups and their replicates. (B) Heatmap showing differential expression of top 200 variable genes between all four groups after filtering genes with low read counts. The color bar indicates *Z* score. (C) Venn diagram of genes overexpressed in each group relative to gRNA only (fold change >2 and padj < 0.05). (D) GO biological process terms of shared genes between the three groups derived from the Venn diagram in (C). Term list was generated using Enrichr; p values were computed using Fisher’s exact test. (E) Expression profiles of select pre-myogenic, myogenic, and satellite cell marker genes from RNA-seq data (mean ± SEM, n = 3 independent replicates). TPM, transcripts per million.

To evaluate the transcriptome-wide gene-expression changes induced by endogenous activation of *PAX7* compared with exogenous cDNA overexpression, we performed RNA-sequencing (RNA-seq) analysis. Differentiated cells that had been treated with either gRNA only, ^VP64^dCas9^VP64^ with gRNA, cDNA encoding *PAX7-A* isoform, or cDNA encoding *PAX7-B* isoform were sorted for mCherry expression at day 14 and RNA was extracted for sequencing. We included *PAX7-B* because it is highly expressed in ^VP64^dCas9^VP64^-treated cells (Figure 2B), yet little is known of its relationship with *PAX7-A*. To gauge the variance between the samples, we generated a sample distance matrix of the RNA-seq data (Figure 6A). This revealed distinct differences between the four treatments, and four unique clusters were readily apparent despite the commonality of induced *PAX7* expression in three of the four groups. Multidimensional scaling of the top 500 differentially expressed genes also showed divergent clustering of sample groups, with *PAX7* cDNA overexpression contributing most to variation between transcriptomic profiles (Figure S3A). We considered the top 200 most variable genes across the four groups and submitted lists of gene clusters apparent on the heatmap for gene ontology (GO) term analysis (Figure 6B). These analyses revealed general developmental pathways including mesoderm development and WNT signaling pathway genes overexpressed in the gRNA-only group. Additionally, this group overexpressed genes involved in heart development such as *HAND1* and *HAND2* (McFadden et al., 2005), which indicates slightly higher propensity of this group to differentiate into cardiac cell lineage. Consistent with this observation, CHIR99021 is also used as the initiator of differentiation of hPSCs into cardiomyocytes (Lian et al., 2012).

GO analyses of genes differentially expressed in the ^VP64^dCas9^VP64^ group were strongly related to myogenesis (Figures 6B and S3B). Genes represented in this group included embryonic myoblast marker *HOXC12* and embryonic MHC *MYH3*, as well as other myogenic regulatory factors *MYOD* and *MYOG*.

Gene set enriched genes following treatment with *PAX7-A* were associated with central nervous system development and NOTCH1 signaling pathways. Interestingly, one of the most differentially upregulated genes in this group was *DLK1* (Figures S3B and S3C), which is required for normal embryonic skeletal muscle development. However, overexpression of *DLK1 in vitro* inhibits proliferation of satellite cells and induces cell-cycle exit and early differentiation (Waddell et al., 2010). Conversely, *Dlk1* knockout increases Pax7^+^ myogenic progenitor cell proliferation *in vitro* and enhances post-natal muscle regeneration *in vivo* (Andersen et al., 2013, Waddell et al., 2010). This would suggest that *DLK1* is involved in maintaining the balance between quiescence and activation of satellite cells. Furthermore, the specific upregulation of both *DLK1* and *DIO3* in these cells (Figures S3B and S3C) suggests activity of the *DLK1-DIO3* gene cluster. This *DLK1-DIO3* locus encodes the largest mammalian megacluster of microRNAs (miRNAs), which is strongly expressed in freshly isolated satellite cells and strongly declined in proliferating satellite cells (Wüst et al., 2018). This decline of *DLK1-DIO3* is concomitant with upregulation of muscle-specific miRNAs, including miR-1, which targets the *PAX7* 3′ UTR to fine-tune its expression and control satellite cell differentiation (Chen et al., 2010). Thus, it is feasible that overexpression of only the *PAX7-A* isoform results in negative feedback and expression of genes and miRNAs that regulate quiescence.

Genes overexpressed specifically in response to *PAX7-B* included brain development genes *VIT* and *OTP*, as well as other *PAX* genes, *PAX2* and *PAX8*, which are involved in kidney development. Although *PAX7* is not implicated in kidney development, CHIR99021 has been used previously to differentiate hPSCs to a kidney lineage (Lam et al., 2014).

Next, we compared each of the three *PAX7*-expressing groups with the gRNA-only group and extracted a list of genes with greater than 2-fold change and adjusted p value (padj) of <0.05 after filtering genes with low read counts. We compared these lists of genes and found that the 56 genes shared in all three groups were enriched for GO terms involved in skeletal muscle development (Figures 6C and 6D). This suggests that compared with treatment with only the gRNA and 14 days of CHIR99021-mediated differentiation, all three groups were able to direct hPSCs into the skeletal myogenic program more effectively than the small-molecule protocol alone. When individual genes are examined, however, the ^VP64^dCas9^VP64^ group outperforms the other groups in terms of expression of pre-myogenic and myogenic genes (Figure 6E). Many of the known satellite cell surface markers and genes are also more highly expressed in the ^VP64^dCas9^VP64^ group compared with the other groups, demonstrating more specific and robust commitment to myogenesis and satellite cell differentiation (Figures 6E and S3D).

## Discussion

In this study, we demonstrate the utility of CRISPR/Cas9-based transcriptional activators for differentiation of hPSCs into MPCs via targeted activation of the endogenous *PAX7* gene. This method serves as an alternative to the transgene overexpression model that has been previously used for myogenic progenitor cell differentiation (Darabi et al., 2012, Kim et al., 2017, Rao et al., 2018). With a minimal small-molecule differentiation protocol involving initial paraxial mesodermal differentiation with CHIR99021 and maintenance with FGF2 in serum-free medium conditions, we demonstrate that targeted activation of the endogenous *PAX7* gene generates a myogenic progenitor cell population that can be passaged at least six times while maintaining PAX7 expression, differentiate readily upon dox withdrawal and subsequent loss of dCas9 activator expression, and engraft into mouse muscle to produce human dystrophin^+^ fibers while also occupying the satellite cell niche. We demonstrate that targeting the endogenous *PAX7* promoter results in enrichment of H3K4me3 and H3K27ac histone modifications, which was sustained for 15 days after dox removal. Enrichment of these chromatin marks was not observed during overexpression of *PAX7* cDNA. Although *PAX7* cDNA overexpression from hPSCs has yielded various degrees of engraftment into NSG mice previously (Darabi et al., 2012, Kim et al., 2017, Magli et al., 2017), we did not obtain similar positive engraftment results with *PAX7* cDNA overexpression under the conditions used here. However, these prior studies used differentiation protocols that generate embryoid bodies, incorporate additional small molecules, or contain animal serum in the medium, and thus differ from the protocol used in this study. Therefore, although our conclusions cannot be extended to other differentiation methods, our findings suggest that activation of the endogenous *PAX7* rather than exogenous *PAX7* cDNA overexpression increases the efficacy of hPSC differentiation into MPCs with robust growth and differentiation potential while retaining regenerative properties following transplantation.

Prior studies using exogenous *PAX7* cDNA relied on overexpression of only the *PAX7-A* isoform (Darabi et al., 2012). However, differential RNA cleavage and polyadenylation yields *PAX7-B*, which contains a highly conserved paired tail domain and is considered to be the canonical sequence (Vorobyov and Horst, 2004). Both isoforms are expressed in human myogenic cells, and orthologs of these PAX7 protein variants are also present in mouse muscle, indicating biological significance for both isoforms. Although distinct functions of these protein variants have not been deciphered, it has been speculated that they may play differential roles in myogenesis that may be necessary for proper satellite stem cell function and myogenic differentiation (Vorobyov and Horst, 2004). Our RNA-seq analysis demonstrates overlapping myogenic function of cells generated by ^VP64^dCas9^VP64^ endogenous activation or *PAX7* cDNA overexpression of either isoforms; however, the ^VP64^dCas9^VP64^ group shared more commonly upregulated genes with *PAX7-B* than *PAX7-A* (89 and 30 genes, respectively), indicating a higher degree of similarity, which is also depicted in the sample distance matrix. The dissimilarity between the overexpression of the two cDNAs indicates that they have distinct functions and can influence global gene expression in separate ways. For example, *PAX7-B* upregulates pre-myogenic genes *PAX3*, *DMRT2*, and satellite cell genes *CXCR4* and *HEY1* more effectively than *PAX7-A*. Conversely, expression of the *DLK1-DIO3* locus that is implicated in satellite cell quiescence is more robust in response to *PAX7-A* than *PAX7-B*. ^VP64^dCas9^VP64^-mediated *PAX7* induction therefore allows expression of both isoforms to properly induce myogenesis at levels of expression that are more likely in the physiological range. Furthermore, endogenous activation of *PAX7* preserves the 3′ UTRs, which are necessary binding targets for the many muscle-specific miRNAs that are known to play a pivotal role in orchestrating proper muscle development and regeneration (Aguilar et al., 2016).

Although conditional expression of *PAX7* in hPSCs via lentiviral transduction is the most promising approach for generating a homogeneous population of engraftable MPCs, integration-free reprogramming will ultimately be preferable for avoiding undesired consequences of genomic integration of viral vectors. ^VP64^dCas9^VP64^ has been demonstrated to rapidly remodel the epigenetic signature of target loci when gRNAs were transiently delivered to achieve neuronal differentiation (Black et al., 2016). In this study, we have also demonstrated that epigenetic signatures are stably maintained in the absence of ^VP64^dCas9^VP64^. In the future, transient delivery of these targeted transcriptional activators via transfection, electroporation, or non-viral nanoparticle delivery of mRNA/gRNA or purified ribonucleoprotein complexes may offer a safer alternative to integration-prone methods.

The expansive CRISPR genome-engineering toolbox offers many possibilities to manipulate cell fates to improve our understanding of the molecular differences between myoblasts, satellite cells, and MPCs generated from hPSCs. Forced transitioning of cell fate still relies on stochastic factors that have remained largely elusive, but generally consist of activation of endogenous networks to generate a stable new identity while also opposing epigenetic memory of the old identity. Further investigation of tissue-specific progenitor cell differentiation from pluripotent cells may unveil fundamental guidelines that will inform a revised model for the generation of a well-defined population of cells capable of repopulating the progenitor cell niche over the long term (Papapetrou, 2016).

In conclusion, these studies introduce a novel method for differentiation and expansion of myogenic progenitors from hPSCs by deterministic editing of transcriptional regulation with new genome-engineering tools, which can enable new possibilities for disease modeling and cell therapy in disorders of skeletal muscle regeneration.

## Experimental Procedures

### gRNA Design, Transfection, and Plasmid Construction

*PAX7* promoter-targeting gRNAs were designed using crispr.mit.edu and cloned into a gRNA vector (Addgene #41824). Candidate *PAX7* gRNAs were transiently transfected with Lipofectamine 3000 on the second day of CHIRON99021-induced differentiation of H9 ESCs constitutively expressing ^VP64^dCas9^VP64^. Cells were harvested after 6 days for qRT-PCR analysis of *PAX7*. For inducible expression, the pLV-hUBC-^VP64^dCas9^VP64^-T2A-GFP plasmid (Addgene plasmid 59791) served as the source vector for generating the pLV-tightTRE-^VP64^dCas9^VP64^-T2A-mCherry. The *PAX7* gRNA was cloned into a pLV-hU6-gRNA-PGK-rtTA3-Blast that was generated using pLV-CMV-rtTA3-Blast as the source vector (Addgene #26429). The *PAX7-A* cDNA (DNASU plasmid HsCD00443491) was cloned into a lentiviral construct to generate pLV-tightTRE-PAX7-P2A-mCherry construct. The *PAX7-A* sequence was confirmed to be the same as the *PAX7* sequence used in previous directed differentiation papers (Darabi et al., 2012). The *PAX7-B* sequence was obtained by PCR of mRNA isolated from cells treated with ^VP64^dCas9^VP64^ + gRNA and cloned into a lentiviral tightTRE-PAX7-B-P2A-mCherry construct.

### Lentiviral Production

HEK293T cells were obtained from the American Tissue Collection Center (ATCC) and purchased through the Duke University Cancer Center Facilities, and were cultured in Dulbecco's modified Eagle's medium (DMEM) (Invitrogen) supplemented with 10% fetal bovine serum (FBS) (Sigma) and 1% penicillin/streptomycin (Invitrogen) at 37°C with 5% CO_2_. Approximately 3.5 million cells were plated per 10-cm tissue-culture polystyrene dish. Twenty-four hours later, the cells were transfected using the calcium phosphate precipitation method with pMD2.G (Addgene #12259) and psPAX2 (Addgene #12260) second-generation envelope and packaging plasmids. The medium was exchanged 12 h post transfection, and the viral supernatant was harvested 24 and 48 h after this medium change. The viral supernatant was pooled and centrifuged at 500 × *g* for 5 min, passed through a 0.45-μm filter, and concentrated to 20× using a Lenti-X Concentrator (Clontech) in accordance with the manufacturer's protocol. Undifferentiated hPSCs were transduced with the pLV-hU6-gRNA-PGK-rtTA3-Blast, and cells were selected with 2 μg/mL of blasticidin (Thermo Fisher Scientific) to generate a homogeneous population of stably transduced cells. Just prior to differentiation, hPSCs were resuspended and plated with lentivirus encoding inducible ^VP64^dCas9^VP64^ or *PAX7* cDNA.

### Cell Culture

H9 ESCs (obtained from the WiCell Stem Cell Bank) and DU11 iPSCs were used for these studies. DU11 iPSCs were generated by the Duke iPSC Shared Resource Facility via episomal reprogramming of BJ fibroblasts from a healthy male newborn (ATCC cell line CRL-2522). Stable and correct karyotype and pluripotency of the cells was confirmed (Rao et al., 2018). hPSCs were maintained in mTeSR (STEMCELL Technologies) and plated on tissue culture-treated plates coated with ES-qualified Matrigel (Corning).

For differentiation, hPSCs were dissociated into single cells with Accutase (STEMCELL) and plated on Matrigel-coated plates at 2.3–3.3 × 10^4^/cm^2^ in mTeSR medium supplemented with 10 μM Y27632 (STEMCELL). The following day, mTeSR medium was replaced with E6 medium supplemented with 10 μM CHIR99021 (Sigma) to initiate mesoderm differentiation. After 2 days, CHIR99021 was removed and cells were maintained in E6 medium with 10 ng/mL FGF2 (Sigma) and 1 μg/mL dox (Sigma).

### Fluorescence-Activated Cell Sorting and Expansion of Sorted Cells

At day 14 after induction of differentiation, cells were dissociated with 0.25% trypsin-EDTA (Thermo Fisher) and washed with neutralizing media (10% FBS in DMEM/F12). Cells were pelleted by centrifugation and resuspended in flow medium (5% FBS in PBS). Cells were sorted for mCherry expression, pelleted, resuspended in growth medium (E6 supplemented with 10 ng/mL FGF2 and 1 μg/mL dox), and plated on Matrigel-coated plates. Cells were passaged every 3–4 days at ∼80% confluency. Terminal differentiation was induced by withdrawing dox from the medium in 100% confluent cultures.

### Flow-Cytometry Analysis

For flow-cytometry analysis of surface markers, cells were harvested during the proliferation phase at day 20 of differentiation. Cells were dissociated with 0.25% trypsin-EDTA, washed with PBS, and resuspended in flow buffer (PBS with 5% FBS). Cells were incubated with the following conjugated antibodies at 0.25 μg/10^6^ cells: immunoglobulin G1-K isotype control-fluorescein isothiocyanate (FITC) (eBioscience 11-4714-41), CD56-FITC (eBioscience 11-0566-41), or CD29-FITC (eBioscience 11-0299-41). Cells were analyzed on a Sony SH800 flow cytometer.

### Cell Transplantation into Immunodeficient Mice

All animal experiments were conducted under protocols approved by the Duke Institutional Animal Care and Use Committee. Seven-week-old female NOD.SCID.gamma mice (Duke CCIF Breeding Core) were used for these *in vivo* studies. Prior to intramuscular cell transplantation, mice were pre-injured with 30 μL of 1.2% BaCl_2_ (Sigma). Twenty-four hours later, MPCs from differentiated iPSCs or ESCs were injected into the TA muscle (5 × 10^5^ cells/15 μL Hank's balanced salt solution). Four weeks after injection, mice were euthanized and the TA muscles were harvested.

### Immunofluorescence Staining of Cultured Cells and Tissue Sections

Cultured cells were plated on autoclaved glass coverslips (1 mm, Thermo Fisher) coated with Matrigel for immunofluorescence staining during the proliferation phase. For differentiation, cells were grown to confluency and differentiated on 24-well tissue culture plates coated with Matrigel, and immunofluorescence staining was performed directly in the well. Cells were fixed with 4% paraformaldehyde (PFA) for 15 min and permeabilized in blocking buffer (PBS supplemented with 3% bovine serum albumin [BSA] and 0.2% Triton X-100) for 1 h at room temperature. Samples were incubated overnight at 4°C with the following antibodies: PAX7 (1:20, Developmental Studies Hybridoma Bank), MHC MF20 (1:200, DSHB), Myf5 (1:200, Santa Cruz Biotechnology sc-302), and MyoD 5.8A (1:200, Santa Cruz sc-32758). Samples were washed with PBS for 15 min and incubated with compatible secondary antibodies diluted 1:500 from Invitrogen and DAPI for 1 h at room temperature. Samples were washed for 15 min with PBS and coverslips were mounted with ProLong Gold Antifade Reagent (Invitrogen), or wells were kept in PBS and imaged using conventional fluorescence microscopy.

Harvested TA muscles were mounted and frozen in optimal cutting temperature compound cooled in liquid nitrogen. Serial 10-μm cryosections were collected. Cryosections were fixed with 2% PFA for 5 min and permeabilized with PBS + 0.2% Triton X-100 for 10 min. Blocking buffer (PBS supplemented with 5% goat serum, 2% BSA, and 0.1% Triton X-100) was applied for 1 h at room temperature. Samples were incubated overnight at 4°C with a combination of the following antibodies: human-specific MANDYS106 (1:200, Sigma MABT827), human-specific Lamin A/C (1:100, Thermo Fisher MA31000), PAX7 (1:10, Developmental Studies Hybridoma Bank), or Laminin (1:200, Sigma L9393). Samples were washed with PBS for 15 min and incubated with compatible secondary antibodies diluted 1:500 from Invitrogen and DAPI for 1 h at room temperature. Samples were washed for 15 min with PBS, and slides were mounted with ProLong Gold Antifade Reagent (Invitrogen) and imaged using conventional fluorescence microscopy.

### Quantitative RT-PCR

RNA was isolated using the RNeasy Plus RNA isolation kit (Qiagen). cDNA was synthesized with the SuperScript VILO cDNA Synthesis Kit (Invitrogen). Real-time PCR using PerfeCTa SYBR Green FastMix (Quanta Biosciences) was performed with the CFX96 Real-Time PCR Detection System (Bio-Rad). The results are expressed as fold-increase expression of the gene of interest normalized to *GAPDH* expression using the ΔΔC_t_ method.

### Chromatin Immunoprecipitation-qPCR

ChIP was performed using the EpiQuik ChIP Kit (EpiGentek) according to manufacturer's instructions. Soluble chromatin was immunoprecipitated with antibodies against H3K27ac and H3K4me3 (Abcam), and genomic DNA (gDNA) was purified for qPCR analysis. All sequences for ChIP-qPCR primers can be found in Table S3. qPCR was performed using PerfeCTa SYBR Green FastMix (Quanta BioSciences), and the data are presented as fold change gDNA relative to negative control (gRNA only) and normalized to a region of the *GAPDH* locus.

### RNA-Seq

RNA was extracted from freshly sorted cells at day 14 of differentiation using the Total RNA Purification Plus Micro Kit (Norgen). Library preparation and sequencing was performed by GENEWIZ on an Illumina HiSeq in the 2 × 150-bp sequencing configuration. All RNA-seq samples were first validated for consistent quality using FastQC v0.11.2 (Babraham Institute). Raw reads were trimmed to remove adapters and bases with average quality score (Q) (Phred33) of <20 using a 4-bp sliding window (SLIDINGWINDOW:4:20) with Trimmomatic v0.32 (Bolger et al., 2014). Trimmed reads were subsequently aligned to the primary assembly of the GRCh38 human genome using STAR v2.4.1a (Dobin et al., 2013) removing alignments containing non-canonical splice junctions (--outFilterIntronMotifs RemoveNoncanonical). Aligned reads were assigned to genes in the GENCODE v19 comprehensive gene annotation (Harrow et al., 2012) using the featureCounts command in the subread package with default settings (v1.4.6-p4) (Liao et al., 2013). The subsequent counts were normalized for each replicate using the R package DESeq2 after filtering out genes that were not sufficiently quantified, and normalized values were used for analysis. Heatmaps were generated using the pheatmap package in R software. Biological processes and pathways were generated using Enrichr (Chen et al., 2013), a web-based online tool. For estimating transcript and gene abundances, transcripts per million were computed using the rsem-calculate-expression function in the RSEM v1.2.21 package (Li and Dewey, 2011).

## Author Contributions

J.B.K. and C.A.G. designed experiments. J.B.K., A.V., A.R.E., and J.D.B. performed the experiments. J.B.K. analyzed the data. J.B.K. and C.A.G. wrote the manuscript.

## Conflicts of Interest

J.B.K. and C.A.G. have filed patent applications related to technologies for genome engineering and cell reprogramming. C.A.G. is a scientific advisor to Sarepta Therapeutics and Iveric Bio, and a co-founder and advisor to Element Genomics and Locus Biosciences.

## Accession Numbers

RNA-seq data (GEO: GSE145575) are available in the Gene Expression Omnibus.

## Supplemental Information

Document S1. Figures S1–S3 and Tables S1 and S2

Document S2. Article plus Supplemental Information
