# Supplementary material for: Myogenic Progenitor Cell Lineage Specification by CRISPR/Cas9-Based Transcriptional Activators
Source: Stem Cell Reports. 2020 Apr 23;14(5):755–69. doi: 10.1016/j.stemcr.2020.03.026 (PMC7221109; doi:10.1016/j.stemcr.2020.03.026)
Supplement: Document S1. Figures S1–S3 and Tables S1 and S2 [file mmc1.pdf]

Stem Cell Reports, Volume *14*

Supplemental Information

Myogenic Progenitor Cell Lineage Specification by CRISPR/Cas9-Based Transcriptional Activators

Jennifer B. Kwon, Ashish Vankara, Adarsh R. Ettyreddy, Joel D. Bohning, and Charles A. Gersbach

Figure S1

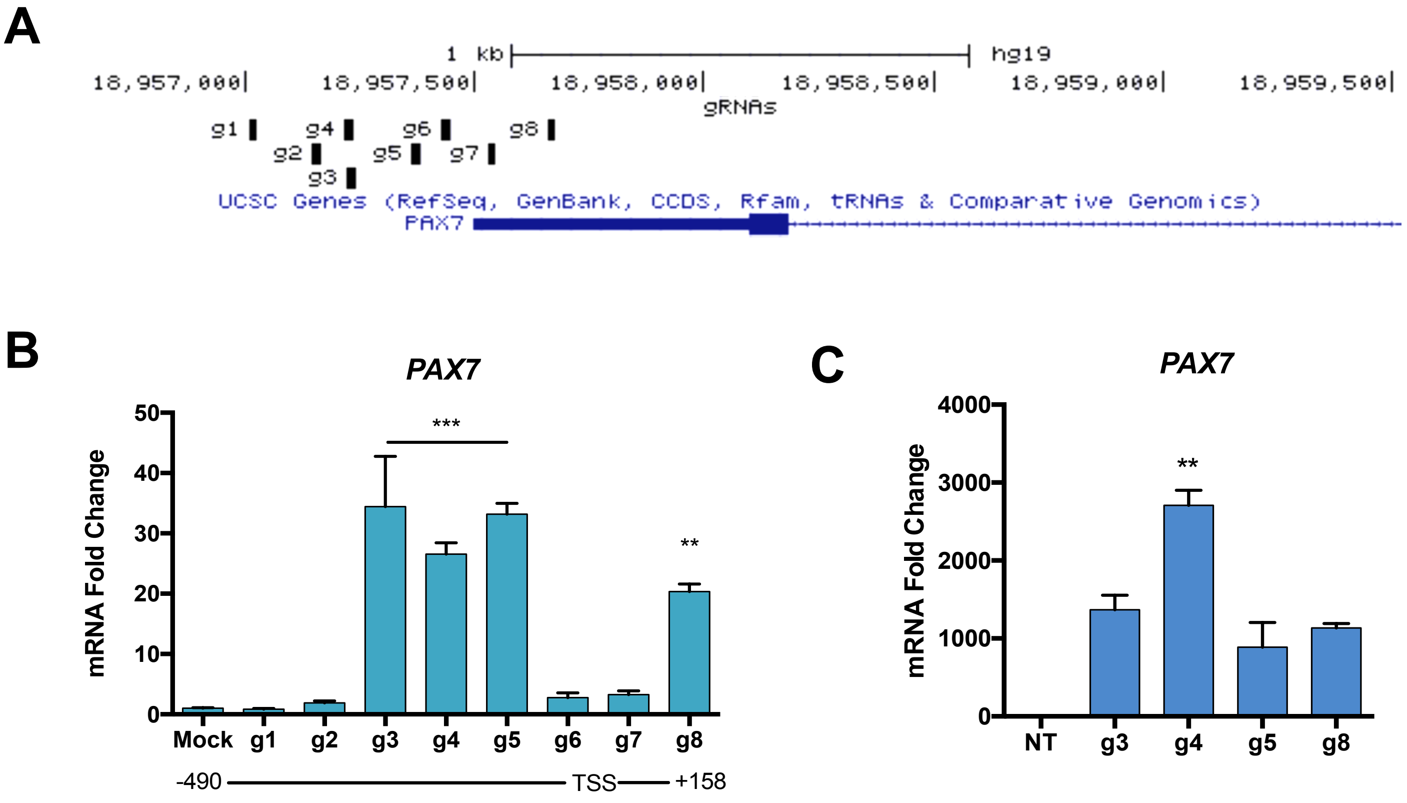

Figure S1. Screening gRNAs for *PAX7* activation with ^{VP64}dCas9^{VP64}, related to Figure 1. (A) gRNA target sites relative to genome browser position of the human *PAX7* gene. (B) Cells expressing ^{VP64}dCas9^{VP64} were treated for two days with CHIRON99021 and lipofected with *PAX7*-targeting gRNAs. Cells were harvested for qRT-PCR analysis after 6 days. gRNA 3, 4, 5 and 8 significantly upregulated *PAX7* compared to mock transfection, but were not significantly different from each other. (C) Lentiviral transduction of gRNAs in paraxial mesoderm cells expressing ^{VP64}dCas9^{VP64} and gRNAs for 1 week. gRNA 4 significantly outperformed the other gRNAs. P values were determined by one-way ANOVA followed by Tukey's post hoc test; $p < 0.05$ (mean ± SEM, n = 3 independent replicates).

Figure S2

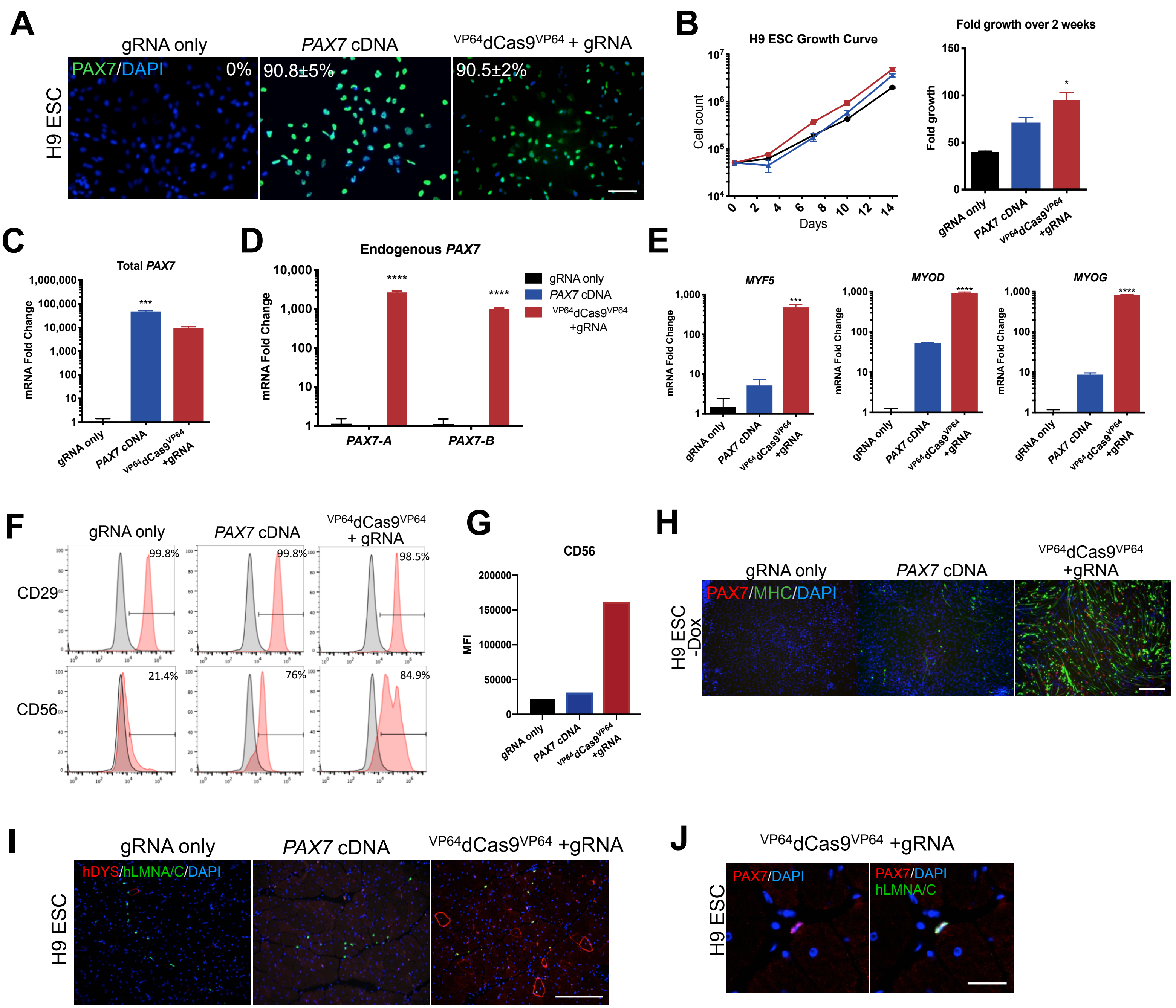

A
gRNA only
PAX7 cDNA
VP64dCas9VP64 + gRNA
PAX7/DAPI
0%
90.8±5%
90.5±2%
H9 ESC
B
H9 ESC Growth Curve
Cell count
Days
Fold growth over 2 weeks
Fold growth
C
Total PAX7
mRNA Fold Change
D
Endogenous PAX7
PAX7-A
PAX7-B
gRNA only
PAX7 cDNA
VP64dCas9VP64 +gRNA
E
MYF5
MYOD
MYOG
F
CD29
CD56
99.8%
99.8%
98.5%
21.4%
76%
84.9%
G
CD56
MFI
H
PAX7/MHC/DAPI
H9 ESC -Dox
I
hDYS/hLMNA/C/DAPI
J
PAX7/DAPI
PAX7/DAPI hLMNA/C

Figure S2. Characterization and transplantation of myogenic progenitors derived from H9 ESCs via ^{VP64}dCas9^{VP64}-mediated activation of endogenous *PAX7* or exogenous *PAX7* cDNA expression, related to Figure 2 and 3. (A) Representative immunostaining of PAX7 at 5 days post-sort. Scale bar = 100 µm. (B) Growth curve of purified myogenic progenitors during post-sort expansion phase was monitored over 2 weeks. (C) Relative amount of total *PAX7* mRNA was determined by qRT-PCR using primers complementary to sequences present in the gene body. (D) Endogenous *PAX7* mRNA was detected using primers complementary to sequencing in the 3' UTR of either *PAX7-A* or *PAX7-B* isoforms. (E) The mRNA expression levels of myogenic markers *MYF5*, *MYOD*, and *MYOG* during the expansion phase. (F) Representative FACS analysis of CD29 and CD56 surface marker expression during the expansion phase. (G) Mean fluorescence intensity (MFI) of CD56 staining intensity across treatments. (H) Representative immunostaining of PAX7 and MHC in differentiated H9 ESCs after 4 passages in the presence of dox. Scale bar = 200 µm. (I) Detection of human-derived fibers in ^{VP64}dCas9^{VP64}-treated cells 1 month after intramuscular injection of $5x10^5$ differentiated ESCs into NSG mice pre-injured with $BaCl_2$. Sections are stained with human-specific dystrophin and lamin A/C antibodies to mark donor-derived fibers and nuclei. Scale bar = 100 µm. (J) Identification of donor-derived satellite cells expressing PAX7 and human-specific lamin A/C. All P values were determined by one-way ANOVA followed by Tukey's post hoc test (mean ± SEM, n = 3 independent replicates). Scale bar = 25 µm.

Figure S3

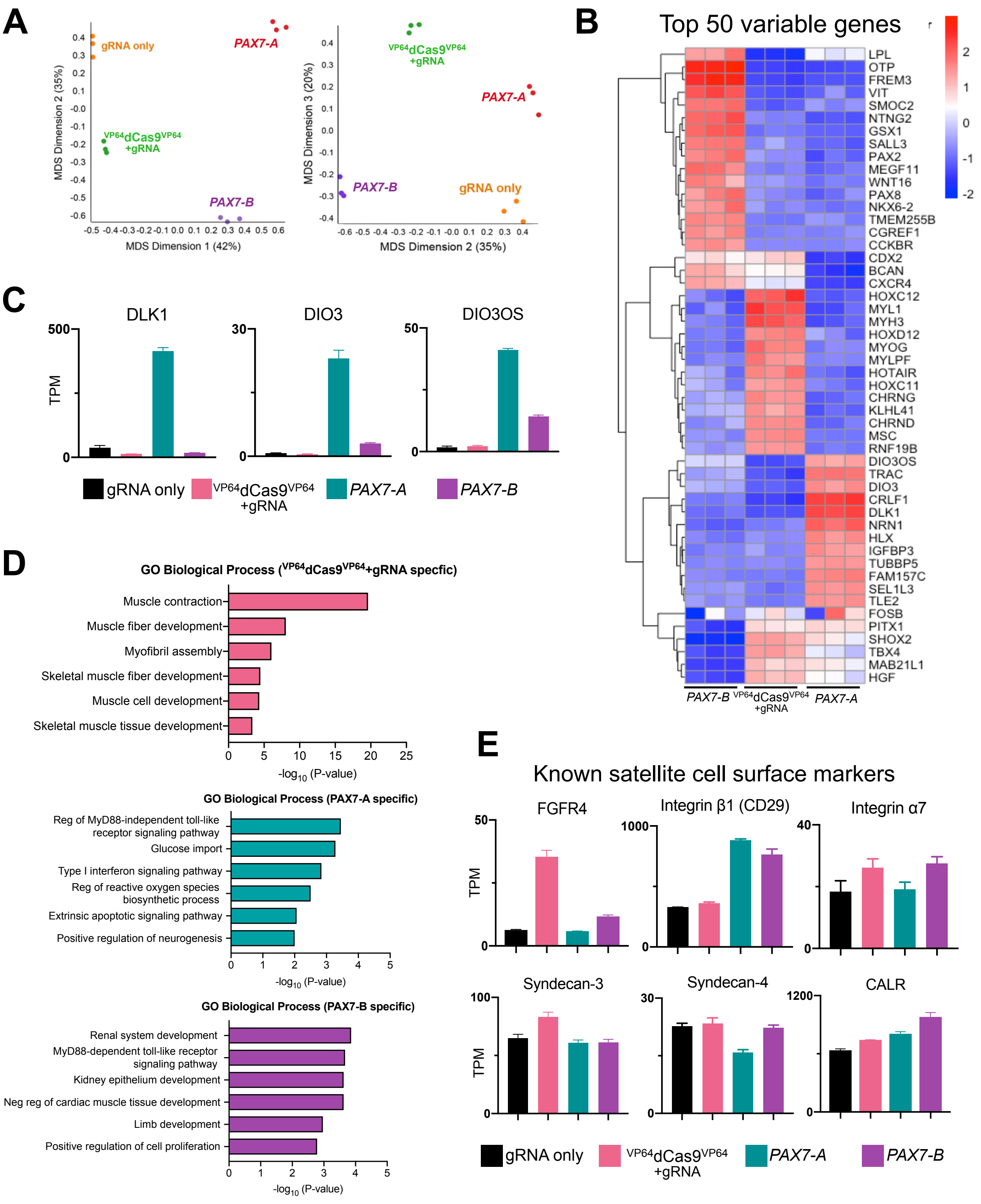
A
gRNA only
PAX7-A
VP64dCas9VP64
+gRNA
PAX7-B
MDS Dimension 1 (42%)
MDS Dimension 2 (35%)
MDS Dimension 3 (20%)
B
Top 50 variable genes
LPL
OTP
FREM3
VIT
SMOC2
NTNG2
GSX1
SALL3
PAX2
MEGF11
WNT16
PAX8
NKX6-2
TMEM255B
CGREF1
CCKBR
CDX2
BCAN
CXCR4
HOXC12
MYL1
MYH3
HOXD12
MYOG
MYLPF
HOTAIR
HOXC11
CHRNG
KLHL41
CHRND
MSC
RNF19B
DIO3OS
TRAC
DIO3
CRLF1
DLK1
NRN1
HLX
IGFBP3
TUBBP5
FAM157C
SEL1L3
TLE2
FOSB
PITX1
SHOX2
TBX4
MAB21L1
HGF
PAX7-B
VP64dCas9VP64
+gRNA
PAX7-A
C
DLK1
DIO3
DIO3OS
TPM
gRNA only
VP64dCas9VP64
+gRNA
PAX7-A
PAX7-B
D
GO Biological Process (VP64dCas9VP64+gRNA specfic)
Muscle contraction
Muscle fiber development
Myofibril assembly
Skeletal muscle fiber development
Muscle cell development
Skeletal muscle tissue development
-log10 (P-value)
GO Biological Process (PAX7-A specific)
Reg of MyD88-independent toll-like receptor signaling pathway
Glucose import
Type I interferon signaling pathway
Reg of reactive oxygen species biosynthetic process
Extrinsic apoptotic signaling pathway
Positive regulation of neurogenesis
-log10 (P-value)
GO Biological Process (PAX7-B specific)
Renal system development
MyD88-dependent toll-like receptor signaling pathway
Kidney epithelium development
Neg reg of cardiac muscle tissue development
Limb development
Positive regulation of cell proliferation
-log10 (P-value)
E
Known satellite cell surface markers
FGFR4
Integrin β1 (CD29)
Integrin α7
Syndecan-3
Syndecan-4
CALR
TPM
gRNA only
VP64dCas9VP64
+gRNA
PAX7-A
PAX7-B

Figure S3. RNA-seq analysis, related to Figure 6. (A) Multidimensional scaling (MDS) of the top 500 differentially expressed genes. (B) Heatmap showing differential expression of top 50 variable genes between the 3 PAX7-expressing groups. The color bar indicates z-score. (C) Expression profile from selected genes overexpressed in response to cDNA encoding *PAX7-A* from RNA-seq (mean ± SEM, n = 3 independent replicates). (D) GO biological process terms for genes specifically enriched in cells treated with ^{VP64}dCas9^{VP64} +gRNA, *PAX7-A* cDNA, or *PAX7-B* cDNA, corresponding to Venn diagram in Figure 4C. (E) Additional expression profiles of known satellite cell surface markers.

Table S1. gRNAs used in this study

gRNA #	Protospacer Sequence (5' - 3')	Position Relative to TSS
1	GGCCGGGGACTCGGCGGATC	-490
2	TCCCCGGCTCGACCTCGTTT	-351
3	CCAGGGCGCAAGGGAGCGG	-278
4	TCCTCCGCTCCCTTGCGCCC	-282
5	GGGGGCGCGAGTGATCAGCT	-137
6	CGGGTTTCAGGGCTGGACGG	-70
7	TGGTCCGGAGAAAGAAGGCG	+30
8	AGCGCCAGAGCGCGAGAGCG	+158

Table S2. qRT-PCR and ChIP-qPCR primers and conditions

Target	Forward Primer (5' - 3')	Reverse Primer (5' - 3')	Cycling Conditions
GAPDH	GAAGGTGAAGGTCGGAGTC	GAAGATGGTGATGGGATTTC	95°C 5s 58°C 20s x40
PAX7	CAGCAAGCCCAGACAGGTGG	GCACGCGGCTAATCGAACTC	95°C 5s 58°C 20s x40
MYF5	AATTTGGGGACGAGTTTGTG	CATGGTGGTGGACTTCCTCT	95°C 5s 58°C 20s x40
MYOD	AGACTGCCAGCACTTTGCTA	GTAGCTCCATATCCTGGCGG	95°C 5s 58°C 20s x40
MYOG	GGTGCCCAGCGAATGC	TGATGCTGTCCACGATGGA	95°C 5s 58°C 20s x40
Endogenous *PAX7-A*	GCTACAAGGTGGTGTCAGGGT	GAGCCATAGTACGGAAGCAGAG	95°C 5s 58°C 20s x40
Endogenous *PAX7-B*	TCTGGCCAAAAATGTGAGCCT	GGGTCAGTTAGGGTTGGGC	95°C 5s 58°C 20s x40
T	TGCTTCCCTGAGACCCAGTT	GATCACTTCTTTCCTTTGCATCAAG	95°C 5s 58°C 20s x40
TBX6	CAACCCCGCATACACCTAGT	CGTCTCGCTCCCTCTTACAG	95°C 5s 58°C 20s x40
MSGN1	AACCTGCGCGAGACTTTCC	ACAGCTGGACAGGGAGAAGA	95°C 5s 58°C 20s x40
PAX3	CTCACCTCAGGTAATGGGACT	CGTGGTGGTAGGTTCCAGAC	95°C 5s 58°C 20s x40
PAX7 ChIP 1 -731bp	CGGGGCTCTGACATTACACA	GCCAGAGTCCGCCCTATTTC	95°C 5s 60°C 20s x40
PAX7 ChIP 2 -289bp	TATTGGTCCTCCGCTCCCTT	GTGAGCGCGATCTGATAGGT	95°C 5s 60°C 20s x40
PAX7 ChIP 3 +562bp	TTGCCGACTTTGGATTCGTC	TCCAAAGGGAATCCCGTGC	95°C 5s 60°C 20s x40
PAX7 ChIP 4 +926	CGCAGGGCTGAAATTCTGGT	AGAGCCGAGAAACTGTCAGG	95°C 5s 60°C 20s x40
